# The Power of Policy: Supporting Latino Aging and Family Care Across Generations

**DOI:** 10.1093/geroni/igaf122.1529

**Published:** 2025-12-31

**Authors:** Jacqueline Angel

**Affiliations:** The University of Texas at Austin, Austin, Texas, United States

## Abstract

For over three decades at The University of Texas at Austin, I have mentored emerging scholars committed to addressing the health and well-being of Latino aging populations. As Wilbur J. Cohen Professor of Health and Social Policy and Mentoring and Principal Investigator of the NIH R13-funded International Conference on Aging in the Americas (ICAA), my mentoring philosophy integrates rigorous training with contextual understanding of policy, culture, and family systems. Through the ICAA’s structured, research-driven mentoring model now embedded in the Texas RCMAR I have guided emerging scholars and early-career underrepresented scholars in successfully publishing in top-tier journals, securing NIH funding (R01, R03, R21, K01), and advancing into academic and policy leadership positions. The ICAA mentorship framework emphasizes cross-national, policy-relevant research using large-scale studies such as the Hispanic EPESE, Mexican Health and Aging Study, and Health and Retirement Study. Recent initiatives include mentoring on pilot projects examining formal and informal caregiving among Medicare-Medicaid dually eligible Latino and non-Latino older adults. At ICAA meetings, I lead program development for juried poster competitions, speed-mentoring sessions, and manuscript development workshops all designed to translate research into policy impact. In this presentation I will reflect on how sustained mentorship rooted in evidence, collaboration, and cultural competency can shape research agendas and policy solutions that promote healthy aging and intergenerational caregiving within Latino communities. Angel has received several recognitions for mentoring research on aging, among which are the 2023 GSA James Jackson Outstanding Mentoring Award, UT Austin Faculty Mentor, President’s Award for Global Learning, 2020. She was elected GSA Fellow in 2000.

